# Understanding maternal nutrition through the voices of community health workers and mother mentors in Limpopo Province, South Africa

**DOI:** 10.3389/fgwh.2026.1850855

**Published:** 2026-07-14

**Authors:** Mxolisi Welcome Ngwenya, Coshiwe Makunyane, Mabitsela Hezekiel Mphasha, Praveenkumar Aivalli, Frank Adusei-Mensah, Samuel Akinseinde, Joke-Folu Badejo, Fionnuala M. McAuliffe, Catherine M. Phillips, Livhuwani Muthelo

**Affiliations:** 1 Department of Nursing Science, University of Limpopo, Mankweng, South Africa; 2 Department of Public Health and Health Systems, University of Limpopo, Mankweng, South Africa; 3 UCD School of Public Health, Physiotherapy and Sports Science, University College Dublin, Dublin 4, Ireland; 4 Institute of Public Health and Clinical Nutrition, School of Medicine, Faculty of Health Sciences, University of Eastern Finland, Kuopio, Finland; 5 UCD Perinatal Research Centre, School of Medicine, University College Dublin, National Maternity Hospital, Dublin, Ireland

**Keywords:** community health workers, health, maternal nutrition, mother mentors, South Africa

## Abstract

**Introduction:**

Maternal nutrition is an important factor influencing maternal health and foetal development. Community health workers (CHWs) and mother mentors (MMs) play a critical role in assisting pregnant women, particularly in low-resource rural settings; however, their knowledge on maternal nutrition remains untapped. This study investigated the attitudes and experiences of CHWs and MMs regarding optimal maternal nutrition among pregnant women in Limpopo Province, South Africa.

**Methods:**

A qualitative exploratory descriptive design was adopted. Purposive sampling was used to recruit participants. Data were collected through in-depth interviews with 11 MMs and five focus group discussions with 34 CHWs. Data were analysed using Braun and Clarke's thematic analysis framework, supported by NVivo and manual coding.

**Results:**

Three themes emerged. The first theme focused on participants’ knowledge and beliefs about maternal nutrition, including food types, dietary behaviours, and the significance of nutrition for maternal and foetal health. The second theme identified barriers to adequate maternal nutrition, such as cultural norms and beliefs, socio-economic constraints, limited resources, misconceptions about pregnancy supplements, and trust concerns. The third theme emphasised the roles of CHWs and MMs, including health education, health literacy promotion, and liaison with healthcare services and community support systems.

**Discussion:**

The findings demonstrate that maternal nutrition habits are influenced by a complex interaction of social, cultural, and economic factors, as well as individual expertise. These findings also highlighted the importance of context-specific and culturally responsive maternal nutrition interventions. Strengthening the competence of CHWs and MMs through standardised training and increased community engagement may contribute to improved maternal nutrition outcomes in disadvantaged settings.

## 1 Introduction

Maternal nutrition is a critical component of maternal and child health, significantly influencing the physical wellbeing, mental health, and quality of life of pregnant women, as well as foetal development (1). Public health efforts—including nutrition interventions and targeted messaging aimed at improving maternal nutrition during pregnancy to mitigate adverse outcomes—are of paramount importance, particularly in low- and middle-income countries (LMICs). Many LMICs face a double burden of malnutrition, comprising both undernutrition and overnutrition, among pregnant women (2–4). Since 2020, the number of malnourished pregnant and breastfeeding women has increased from 5.5 million to 6.9 million (4). This burden disproportionately affects LMICs, particularly countries such as Nigeria, Sudan, Yemen, Burkina Faso, Kenya, Mali, Niger, Ethiopia, Chad, and Somalia (5). Furthermore, there is evidence of considerable urban–rural disparities in Sub-Saharan Africa, where more than two-thirds of pregnant women in rural areas are malnourished (6).

Pregnancy complications, including hypertensive disorders and obstetric haemorrhage, account for more than half of maternal deaths in South Africa (7). Existing evidence from LMICs indicates that micronutrient inadequacy during pregnancy is associated with an increased risk of complications (8). In support of this, O’Nan et al. highlighted that poor dietary patterns are a modifiable risk factor for hypertensive disorders in pregnancy, noting that diets high in dairy products, saturated fat, and sodium predispose women to developing hypertensive disorders during pregnancy (9).

The South African Addendum to the Maternal, Perinatal, and Neonatal Health Policy highlights a critical gap in the integration of maternal nutrition into the continuum of maternal healthcare services, a challenge further exacerbated by shortages in human resources and limited staff capacity (10). To address the growing healthcare demands, South Africa has undertaken a reengineering of primary healthcare, which includes task shifting to community health workers (CHWs) and mother mentors (MMs). These cadres provide guidance and support to pregnant women throughout pregnancy, including during antenatal care visits, thereby improving access to maternal healthcare services (11). CHWs and MMs act as paraprofessionals employed by the Department of Health, serving as intermediaries between the healthcare system and communities. They deliver preventive, promotive, and supportive community-based services to pregnant women and their families (12).

Despite these efforts, in Limpopo Province—one of the most rural provinces in South Africa—there remains a paucity of data on the perceptions and understanding of CHWs and Mother Mentors regarding maternal nutrition. Given that CHWs, supported by MMs, are at the forefront of healthcare delivery in rural and underserved areas, understanding their perspectives is essential to inform strategies that promote optimal maternal nutrition and improve maternal and perinatal outcomes. This study, therefore, explores the understanding and insights of CHWs and MMs regarding maternal nutrition among pregnant women in Limpopo Province. Guided by the Health Belief Model (HBM), the study examines perceived severity, barriers, benefits, threats, modifiable factors, and cues to action related to maternal nutrition among pregnant women in South Africa, from the perspectives of CHWs and MMs.

### 1.1 Conceptual framework: Health Belief Model

The HBM is a popular theoretical framework for analysing health-related behaviours, particularly in preventative health settings. It contends that individuals’ participation in health actions is influenced by their ideas about a condition and the perceived benefit of acting. The HBM's core dimensions are perceived advantages, which pertain to perceptions about the efficacy of a health practice; perceived obstacles, which include social, cultural, and economic limitations; and perceived dangers, which represent an individual's estimate of susceptibility and severity. In addition, cues to action serve as internal or external triggers that motivate behavioural change (13). In maternal health, the HBM provides a useful lens for examining how pregnant women interpret and respond to nutritional guidance. It also enables exploration of how frontline actors, such as CHWs and MMs, influence health behaviours. In this study, the HBM informed the organisation and interpretation of findings related to maternal nutrition.

## 2 Methods

### 2.1 Study design

This study utilised a qualitative descriptive design with thematic content analysis. The relevant institutional review board granted ethical approval, and all participants provided written informed consent before data collection.

The qualitative descriptive approach was used to investigate and provide a comprehensive account of the understanding and insights of CHWs and MMs regarding maternal nutrition in South Africa. In addition, the study examined perceived barriers and threats that influence pregnant women's dietary practices. The study is reported in accordance with the Consolidated Criteria for Reporting Qualitative Research (COREQ) guidelines (14), with the completed COREQ checklist provided as Supplementary Material.

### 2.2 Setting

The study was conducted in Limpopo Province, the northern province of South Africa, with a focus on the Capricorn District. Most participants were drawn from rural settings, reflecting the predominantly rural composition of the province, while a small proportion (4 out of 34 participants) were from semi-urban areas. The study focused on rural CHWs and MMs, who constituted the large majority of the sample. A small number of MMs worked in semi-urban or urban settings. Their accounts were included to reflect the diversity of participant experiences; however, the small number of semi-urban participants was insufficient to support any formal comparison across rural and semi-urban contexts. Institutional representation in this study was largely rural, with 7 of the 11 participating institutions located in rural areas. All 34 CHWs were from rural settings. Of the 11 MMs, seven worked in rural areas and four represented semi-urban or urban contexts (Table 1). This distribution reflects the demographic reality of the Capricorn District, where more than 65% of the population resides in rural areas, thereby justifying the emphasis on rural perspectives.

**Table 1:** Demographic data of the CHWs and MMs.

Attributes	CHW (*n* = 34)	MMs (*n* = 11)
Age group, *n* (%)		
• 36–45 years	5 (14.7)	3 (27.3)
• 46–55 years	18 (52.9)	8 (72.7)
• 56–65 years	11 (32.3)	0 (0)
Gender		
• Female, *n* (%)	34 (100)	11 (100)
• Male	0 (0)	0 (0)
Workplace location		
• Rural, *n* (%)	34 (100)	7 (63.6)
• Urban, *n* (%)	0 (0)	4 (36.4)
Trained in maternal nutrition, yes, *n* (%)	4 (11.8)	2 (18.2)

Limpopo Province has one of the highest maternal death rates in South Africa, ranking fourth overall. The province is also marked by high rates of poverty (56.0%) and unemployment, particularly in isolated rural areas. These socio-economic problems contribute to limited access to healthcare services, linked to poor maternal and perinatal outcomes. The problem is compounded by a dearth of healthcare workers (15). The Capricorn area, an important area in the province, is distinguished by its cultural diversity and expanding infrastructure, which includes both rural and semi-urban communities. The sample's primarily rural character reflects this demographic reality and provides an appropriate focus for the study, given that CHWs in the area work almost exclusively in rural communities. The location was thus deemed adequate for achieving the study objectives.

### 2.3 Recruitment and sampling

Data were collected from a total of 11 primary healthcare facilities, seven located in rural areas and four in semi-urban areas. The study included five focus group discussions (FGDs), primarily with CHWs, and 11 in-depth individual interviews conducted with MMs. The study employed heterogeneous purposive sampling to capture a broad range of perspectives and diverse insights from CHWs and MMs regarding maternal nutrition. This sampling strategy was intended to capture a broad range of experiences among CHWs and MMs, who worked predominantly in rural settings. CHWs affiliated with the selected primary healthcare facilities provide household-level maternal health services to pregnant women, while MMs are facility-based and offer complementary health education, guidance, support, and mentorship during antenatal visits. Participants were purposively selected from 11 primary healthcare facilities based on their active involvement in delivering maternal health services; the CHWs worked exclusively in rural communities, while the MMs were facility-based, with a small number serving semi-urban or urban areas.

Following institutional permission from the participating facilities, the primary author and trained research assistants conducted participant recruitment. Eligible CHWs and MMs who were present at the facilities on the day of data collection were invited to assemble in a private room. The purpose of the study, methodology, and ethical considerations were clearly explained to all potential participants prior to enrolment. Inclusion criteria required CHWs and MMs to be employed by the Department of Health in accordance with national policy guidelines, with at least one year of professional experience and active engagement in providing maternal health services to pregnant women. This ensured that participants had sufficient experience to provide informed insights relevant to the study objectives. Participation was entirely voluntary. Those who agreed to participate provided written informed consent for data collection. There were no refusals, and no participants withdrew from the study.

Sample size was guided by thematic saturation, encompassing both code saturation (the point at which no new codes emerged) and meaning saturation (the point at which no new insights or dimensions of existing themes were identified). Code saturation was initially reached after three FGDs for CHWs and eight in-depth interviews with MMs. Additional FGDs and interviews were conducted to confirm meaning saturation, as no new codes, themes, or interpretive insights emerged. Saturation was further supported through concurrent transcription, iterative coding, and constant comparison of categories across the dataset.

### 2.4 Data collection

Data were collected using a combination of FGDs and individual semi-structured interviews, guided by an interview protocol developed by the research team. The interview guides were informed by existing literature on maternal nutrition and were designed to elicit in-depth perspectives, experiences, and insights from participants. The following were some of the key questions: “What is your understanding of maternal nutrition?” “What are your roles and responsibilities in supporting pregnant women with nutrition in your community?” The interview guides were pilot tested in a non-participating clinic to assess clarity, relevance, and flow. Following the pilot, the guides were reviewed and refined by co-authors with expertise in qualitative research and maternal health. Minor revisions were made, including the removal of redundant questions and refinement of wording to enhance clarity and depth of responses. Data collection was conducted under the supervision of an experienced female professor. The field team comprised the primary author, an experienced male qualitative researcher, two female research assistants with master's-level training, and a female postdoctoral researcher. Prior to data collection, the primary author facilitated a refresher training session for the research team, focusing on qualitative research methods, ethical considerations, and best practices for conducting FGDs and interviews. Data collection took place between April and July 2025. Interviews and FGDs were conducted in participants’ preferred language (Sepedi or English) to facilitate rich and authentic responses. All members of the research team were fluent in both languages, and interviews conducted in Sepedi were transcribed and translated into English. Each FGD and interview lasted approximately 60–90 min.

All sessions were conducted in private rooms within the participating clinics to ensure confidentiality and minimise interruptions. During data collection, the researchers employed reflexive techniques, including bracketing, to acknowledge and set aside personal biases and preconceptions. Efforts were also made to build rapport with participants by clearly introducing the research team, explaining their roles, and outlining the purpose and significance of the study. This approach fostered a comfortable environment, enabling participants to engage openly and provide detailed responses.

### 2.5 Data analysis

Data were analysed using the thematic analysis framework initially developed by Braun and Clarke, employing a hybrid approach that combined inductive and deductive strategies (16). Initially, an inductive approach was applied, allowing themes to emerge directly from the data through the six iterative phases of thematic analysis: familiarisation, coding, theme generation, theme review, theme definition, and reporting. This approach enabled an in-depth exploration of participants’ understanding and experiences of maternal nutrition.

Subsequently, a deductive approach was used to map the emerging themes onto the constructs of the HBM, including perceived susceptibility, severity, benefits, barriers, and cues to action. This theoretical framing facilitated a structured interpretation of how CHWs and MMs understand maternal nutrition and the factors influencing pregnant women's dietary practices.

Audio recordings from interviews and FGDs were transcribed verbatim. Interviews conducted in Sepedi were translated into English by members of the research team who were fluent in both languages. The translated transcripts were cross-checked by bilingual team members to ensure accuracy. Although no independent translator was employed, the research team's familiarity with the local cultural and linguistic context enabled preservation of semantic, contextual, and cultural meanings, prioritising conceptual equivalence over literal translation.

All transcripts were read multiple times to ensure data familiarisation. Initial codes were developed and documented in a codebook, informed by both the interview guide and emerging data. These codes were systematically organised into potential themes that reflected CHWs’ and MMs’ understanding and perceptions of maternal nutrition. Themes were iteratively reviewed against the coded data and refined to ensure internal coherence and accurate representation of participants’ accounts.

Data management and analysis were supported by NVivo version 12 software, alongside manual coding processes, to enhance rigour and ensure systematic organisation of the data. Two researchers (MWN and PA) independently conducted coding and initial theme development. Regular meetings were held to compare coding decisions and discuss discrepancies. Consensus on the final themes was achieved through team discussions, with the initial meeting focusing on code comparison and the second on final theme refinement after thorough data review. All identified themes were aligned with the constructs of the Health Belief Model, and no themes emerged outside the scope of the theoretical framework (16).

### 2.6 Trustworthiness

Trustworthiness was ensured using the criteria proposed by Alexander (17) encompassing credibility, dependability, confirmability, and transferability.

**Credibility** was established through prolonged engagement with participants during data collection, with interviews and focus group discussions lasting approximately 60–90 min over one month. This facilitated the collection of rich and in-depth insights into maternal nutrition. Field notes were maintained during all sessions to capture nonverbal cues and contextual information. The research team also established rapport with participants during the recruitment phase to promote openness and minimise potential response bias. Credibility was further strengthened through iterative data familiarisation, involving repeated reading of transcripts and listening to audio recordings. Data were cross-referenced to ensure a comprehensive and accurate interpretation of participants’ perspectives. In addition, member checking was conducted through a feedback workshop with selected participants, during which preliminary findings were presented and discussed to validate the accuracy and relevance of the interpretations.

**Dependability** was enhanced through methodological rigour and transparency. Triangulation of data collection methods (FGDs and individual interviews) was employed to reduce bias and improve consistency. Furthermore, analyst triangulation was achieved by having two researchers (MWN and PA) independently code the data. Coding decisions were compared and refined through discussion, and discrepancies were resolved collaboratively among the research team to ensure consistency in interpretations (18).

**Confirmability** was ensured through peer debriefing and collaborative analysis involving all authors. The findings were grounded in participants’ verbatim quotations, thereby minimising researcher bias and enhancing objectivity. An audit trail was maintained throughout the study, documenting methodological decisions, coding processes, and analytical steps, allowing for transparency and reproducibility of the research process.

**Transferability** was supported through purposive sampling of CHWs and MMS and through a detailed description of the study context, participants, and predominantly rural procedures, which were provided to enable readers to assess the applicability of the findings to similar settings. The limited semi-urban representation and the absence of any urban CHWs constrain transferability to non-rural settings. The research team comprised individuals with expertise in public health, maternal health, and nutrition, who had no prior relationships with the CHWs and MMs before the study commenced. Reflexivity was maintained throughout the research process, including the use of bracketing to acknowledge and minimise preconceptions. Additional strategies to reduce researcher bias included strict adherence to the interview guide, co-coding of data, and ongoing peer debriefing.

## 3 Results

A total of 45 participants were included in the study, comprising 34 CHWs who participated in focus group discussions and 11 MMs who participated in individual interviews. All participants were recruited from primary healthcare facilities within the Capricorn District, Limpopo Province. Both CHWs and MMs were originally employed by nongovernmental organisations. At the time of data collection, both cadres had been integrated into government services. Their roles and training pathways differed. MMs had previously worked alongside nurses in clinics and communities, primarily supporting pregnant women living with HIV. CHWs were part of a government-led community-oriented primary care model.

Training duration and content varied between the two groups. MMs reported completing either an 8-week training programme or a combined 4-week classroom and 2-week field-based training. This training included components on HIV, nutrition, alcohol use, and mental health. In contrast, CHWs reported receiving shorter and less standardised training, with variation in duration and content across regions. Participants from both groups reported having educational attainment below that of professional nurses, with most having completed secondary education (Matric level). MMs were selected based on their lived experience as mothers living with HIV. Demographic characteristics of CHWs and MMs are presented in Table 1. All participants were female. Among CHWs, 52.9% were aged 46–55 years, and all were based in rural areas. Among MMs, 85.7% were aged 46–55 years, and 63.6% were based in rural settings.

### 3.1 Themes and subthemes

Thematic analysis identified three major themes: (i) understanding and perceptions of maternal nutrition, (ii) barriers and challenges to achieving optimal maternal nutrition among pregnant women, and (iii) roles of CHWs and MMs in supporting maternal nutrition. These themes were supported by nine subthemes, all of which are presented in Table 2. The findings were interpreted using the Health Belief Model to systematically examine how perceived risks, benefits, barriers, cues to action, and self-efficacy shape maternal nutrition behaviours.

**Table 2:** Themes and subthemes based on the HBM.

HBM construct	Theme	Subtheme	How the construct is reflected
Perceived susceptibility	Barriers and challenges	Cultural practices and beliefs	Beliefs about vulnerability shaped by fear of harm, bewitchment, or social norms
Perceived severity	Understanding and perceptions	Awareness of maternal nutrition	Recognition of consequences such as poor foetal development and maternal illness
Perceived benefits	Understanding and perceptions	Dietary practices and recommendations	Beliefs that good nutrition improves foetal growth, blood production, and immunity
Perceived barriers	Barriers and challenges	Poverty and resource constraints	Limited affordability, food insecurity, and lack of access to services
Perceived barriers	Barriers and challenges	Misconceptions about supplements	Beliefs that supplements cause large babies or harm
Perceived barriers	Barriers and challenges	Lack of trust in CHWs and MMs	Reduced uptake of advice due to credibility issues
Cues to action	Role of CHWs and MMs	Nutrition and hygiene education	Health education prompts behaviour change
Cues to action	Role of CHWs and MMs	Health literacy activities	Encouragement of antenatal care, lifestyle advice
Self-efficacy	Role of CHWs and MMs	Liaison and support services	Referral systems and support mechanisms enabling action

#### 3.1.1 Theme 1: Understanding and perceptions of maternal nutrition

The findings revealed that CHWs and MMs demonstrated differing levels of knowledge and understanding of maternal nutrition. Participants described nutrition-related concepts, identified key food groups, and outlined approaches to daily meal planning during pregnancy. They also emphasised the role of maternal nutrition in supporting maternal health and foetal development. The reported benefits of optimal maternal nutrition included support for foetal growth and blood production. Two subthemes were identified from the data: (1) knowledge and awareness of maternal nutrition, and (2) dietary practices and recommendations. These subthemes are presented in the following with illustrative participant accounts.

##### 3.1.1.1 Subtheme 1.1: Perceptive insight and awareness of maternal nutrition

Although most CHWs and MMs reported not having received formal training in maternal nutrition, many demonstrated knowledge and awareness of key aspects of maternal nutrition. Participants described maternal nutrition as important for both the mother and the foetus, identifying its role in supporting foetal growth and maternal immunity. Similar descriptions were provided by both rural and urban MMs.

These findings are illustrated by the following participant quotes:

“Maternal nutrition helps the unborn baby grow well.” FDG 3, P1 (Facility 1)

“It also helps the pregnant woman to be healthy without sicknesses during pregnancy.” FDG 3, P2 (Facility 1)

“When a woman is pregnant, she is eating for both herself and the unborn child, so she needs to eat the correct food, which will assist in the growth of the baby, because if the mother is not eating right, the baby might be underdeveloped. My understanding is that we will continue encouraging every pregnant woman to eat well for the development of the baby.” FGD 5, P1 (Facility 6)

“I think the importance is that when we say a pregnant woman should eat healthy food, it's because the baby is also going to eat what the mother consumes, and it's for the baby to be well developed and delivered in a healthy state. I forgot to mention that a pregnant woman should drink enough water.” FGD 4, P2 (Facility 8)

“I understand that they help a pregnant woman be healthy. Like, if you eat vegetables, you will have increased blood production. Some foods reduce the likelihood of being infected by diseases while pregnant. Like oranges have vitamin C that assists in fighting conditions like the flu.” P2, Mother mentor (Facility 2)

##### 3.1.1.2 Subtheme 1.2: Dietary practices and recommendations

Participants described common dietary practices among pregnant women in their communities, including the types of foods and beverages consumed. Several participants reported that pregnant women frequently consumed foods such as kota, chips, and sugar-sweetened beverages. In response to these practices, both CHWs and MMs reported advising pregnant women to limit the consumption of such foods. They described recommending a balanced diet consisting of a variety of food groups, including vegetables, staple carbohydrates, meat, milk, eggs, and legumes. Participants also emphasised the inclusion of locally available foods, such as home-grown produce, in the dietary guidance provided to support maternal nutrition:

“Ooh, we encourage pregnant women to eat food such as beans, carrots, cabbage and food that they should eat, which is healthy foods, and we discourage them from eating soil, smoking and taking drugs. We discourage them from eating unhealthy Food like chips and fatty cakes/unhealthy food.” FDG 1, P2 (Facility 1)

“…food that contains protein because they need to have enough blood, especially during delivery and food like spinach, livers and eggs. I believe the food will help as it does not have too much fat, and with less salt, it will assist them to have enough blood to avoid a situation whereby they become anaemic at birth.” FDG 5, P6 (Facility 6)

“Firstly, I advise them to eat food that contains carbohydrates, which gives energy. This food contains starch and its pap, rice & samp. The second category of food that I advise them to eat is protein. Proteins are. Proteins are foods that build the body, and they can be white meat (fish or chicken) and red meat (beef, goat, and sheep). The third category is vitamins, mostly fruits and vegetables….” P8, Mother mentor (Facility 5)

Alongside recommendations for an optimal maternal diet, CHWs and MMs emphasised the importance of complementary practices such as exercise and drinking plenty of water. The participants viewed exercise as pivotal for pregnant women, as it would assist them during the childbirth process. For instance, the participants added the following:

“We advise them to exercise and drink enough water. A person is not supposed to just eat and sleep. The baby might be born sleeping or finish the whole week sleeping, but if the mother exercises by just stretching or doing light chores and drinks enough water, all will be well.” FGD 4, P2 (Facility 8)

“We advise our pregnant women to constantly drink water and to exercise, and they must not eat fatty food…” FGD 1, P6 (Facility 1)

“Food that our pregnant women must eat is fruits, vegetables and food like proteins such as fish, and they must drink a lot of water. You are not to eat, and be lazy around. One must exercise when pregnant so that they can use the energy and for the child to be active.” FGD 1, P3 (Facility 1)

Participants provided differing accounts regarding advice given to pregnant women about physical activity. Some participants described recommending exercise during pregnancy, including explanations that it may influence infant outcomes, such as preventing a baby from being “sleepy” at birth.

#### 3.1.2 Theme 2: Barriers and challenges to achieving optimal maternal nutrition among pregnant women

When describing challenges related to achieving optimal maternal nutrition among pregnant women, CHWs and MMs referred to a range of factors, including cultural practices and beliefs, availability of resources, and socio-economic conditions. Participants described how these factors influenced food choices and the ability of pregnant women to follow a balanced diet. Four subthemes were identified and are presented in the following.

##### 3.1.2.1 Subtheme 2.1: Cultural practices, myths, and beliefs around maternal nutrition

In this subtheme, CHWs and MMs described experiences and observations related to cultural practices, beliefs, and norms influencing maternal nutrition. Participants reported that some pregnant women used herbal remedies and sought guidance from traditional or religious practitioners during pregnancy.

They also described dietary restrictions associated with cultural practices, where certain foods were avoided during pregnancy. These accounts illustrate how cultural beliefs and practices shape dietary behaviours among pregnant women in the study settings. These findings are illustrated by the following participant narratives:

“They say a pregnant woman should not eat tripe because the baby will be born without hair. Drinking Fanta is also prohibited because the baby's eyes will be yellow.” FGD 1, P2 (Facility 1)

“We do encounter such situations. For instance, a pregnant woman might share that a prophet or spiritual leader has advised her to avoid consuming salt and instead drink plenty of water or follow other specific practices. We often hear stories from pregnant women about cultural or traditional practices they're following, such as visiting traditional healers for guidance on pregnancy-related issues.” P1, Mother mentor (Facility 1)

“Eh, there is a thing called Chikwana, it looks like black ash. Most of the time, they make pregnant women take it before coming to the clinic to prevent evil spirits because at the clinic, it is believed that they are going to be amongst a lot of people….” P4, Mother mentor (Facility 1)

Cultural practices, myths, and beliefs were described as present in both urban and rural areas of the study setting. Participants reported that these practices influenced dietary behaviours during pregnancy, including restrictions on certain foods. They also indicated that cultural beliefs affected healthcare-seeking behaviours, with some pregnant women delaying access to healthcare services due to fears related to bewitchment. Food taboos were commonly reported across communities and were described as shaping nutritional practices during pregnancy. CHWs and MMs reported engaging with pregnant women to provide clarification and information regarding these beliefs:

“We have had women who refuse to go to the clinic because they hide their pregnancy from the witches… We keep teaching them about the importance of going to the clinic when pregnant.” FGD 3, P2 (Facility 1)

“Some women are secretive about pregnancy because, culturally, you must hide pregnancy. They don't go to the clinic ….” FGD3, P4 (Facility 1)

##### 3.1.2.2 Subtheme 2.2: Resource constraints and poverty

Participants from both rural and urban settings described socio-economic conditions as influencing the ability of pregnant women to achieve optimal maternal nutrition. Affordability of food was commonly reported as a key factor. CHWs and MMs indicated that many pregnant women were unemployed and unmarried, which limited their financial resources for purchasing nutritious foods.

Participants further reported that some pregnant women relied on extended family members, including elders and grandparents, as their primary source of food. In addition to financial constraints, participants described limited access to supportive resources. These included insufficient availability of food support for vulnerable households, limited access to social grants, and a lack of structured referral pathways linking pregnant women to community-based support services. These factors were described alongside existing health education efforts:

“You know, you find that in the family, there is a parent and no one is working in that household with 3 kids and the grant money they get is prioritised on paying policies, electricity, and to buy basic food for the kids to eat before going to school in the morning. In addition to that, if the parent gets pregnant again, it becomes a very big challenge, and they can't even afford healthy food because the priority would be looking at buy toiletries and electricity. The fruits and healthy food are not affordable in that situation….” FGD1, P1 (Facility 1)

“Most pregnant women are unemployed, and if you are pregnant, staying with an elderly woman/granny, they normally would say to a pregnant woman that ‘I won't be able to feed with essential food like potatoes and cabbage….’” FGD4, P2 (Facility8)

“…unemployment as some depend on the social grants of their children.” FGD2, P4 (Facility 1)

“We try, but unfortunately, because of poverty in some houses, we end up not talking about food.” FGD3, P3 (Facility 1)

“What I understand as a contributing factor for them not to eat healthy food is a lack of water and money to buy this and that….” P7, Mother mentor (Facility 4)

The study findings further identified that in an attempt to overcome these challenges, the participants reported advising these pregnant women to plant vegetables within their yard. However, challenges persisted, particularly in urban areas where pregnant women highlighted that the yard space is too small:

“…We keep telling them to try to eat healthy. When we tell them to plant vegetables, they say they stay in town, they do not have enough land.” P10, Mother mentor (Facility 7)

##### 3.1.2.3 Subtheme 2.3: Misconceptions and myths on pregnancy supplements

Participants described challenges related to the use of nutritional supplements during pregnancy. CHWs and MMs reported that supplements such as folic acid, calcium, and ferrous sulphate were routinely provided by nurses to support maternal nutritional needs. However, participants indicated that some pregnant women do not consistently take these supplements. Reported reasons included beliefs that supplement use may lead to increased foetal size. Participants also described limited formal guidance on how to address such beliefs. CHWs and MMs indicated that, in these situations, they rely on personal experience, individual knowledge, or informal peer support when engaging with pregnant women about supplement use. The CHWs stated the following:

“Problem with our people is that when you advise them to take the supplements given at the clinic, they say the pills makes the tummy to grow bigger. They still have the mindset of saying if we drink pills from the clinic, we will have big tummies….” FGD 2, P5 (Facility 1)

“…Our duty is just to encourage them to take their given supplements daily because others just take them and do not drink because they might lack knowledge on the importance of those supplements.” FGD5, P1 (Facility 6)

In support of this, the mother mentor indicated, “We encourage them to drink the folic acid and calcium that they are given here at the clinic. This is because there is a myth that the supplements make the baby grow bigger. We make them aware that the supplements aid in the well development of the baby…but, some myths are rooted in their heads, just like those.” P8, Mother mentor (Facility 5)

##### 3.1.2.4 Subtheme 2.4: Lack of trust toward CHWs and MMs

Participants described perceived challenges related to how their roles and services were received within communities. CHWs and MMs reported that their advice, including guidance on maternal nutrition, was not always followed. They indicated that some community members viewed their roles as limited and expressed reluctance to engage with the information provided.

Participants further reported that pregnant women and other community members often placed greater trust in nurses, as well as in informal sources such as family members and peers, when making decisions related to nutrition and health during pregnancy:

“…to be honest, what I have realised about our job in the community, people undermine what we do and what we see, household to household, is heartbreaking.” FGD1, P1 (Facility 1)

“Sometimes they undermine us because we are just community healthcare workers; they need the nurses to talk to them about maternal nutrition….” FGD3, P6 (Facility1)

“…it's difficult for us to reach out the way we want, especially since we are working in the same community where we live, and they don't feel comfortable….” FGD5, P2 (Facility 6)

“Others will tell you that you can't tell them what to eat because they are grown-ups and have been eating since childhood. So, if that happens, I don't get angry because I know that people are not the same….” P6, Mother mentor (Facility 4)

The participants further indicated that instead of taking advice from them on maternal nutrition, the pregnant women chose to follow what they learned from their peers, friends, grandmothers, and social media:

“They have friends and families who talk to them before we even come; by the time we reach, they are clouded by the myths related to maternal nutrition.” FGD3, P5 (Facility 1)

“They get them from their households and from the street. Most of them are staying with elderly people….” P9, Mother mentor (Facility 6)

##### 3.1.2.5 Subtheme 3.1: Nutrition and hygiene education

Despite reporting limited formal training in maternal nutrition, participants described their role as primarily focused on providing health education. This included advising on the types and variety of foods recommended during pregnancy:

“We advise them to eat healthy food like vegetables and fruits. Before they eat fruits, they must wash them and not overindulge because of availability, e.g., if it's a green apple, we advise eating once (1) per day. They must eat cereal like Weetbix and cornflakes so that the cereal can absorb fat in the body, and for them to frequently drink water and avoid eating too much salt.” FGD4, P4 (Facility 8)

“We encourage pregnant women to eat beetroot because it increases the amount of blood in the body. They should eat spinach, cabbage, fruits, and must also stay in a clean environment and bathe regularly. We now give them iron supplements at the clinic; they must drink them so that the baby can be healthy. When a woman is pregnant, not only is she eating for herself, but she is also eating for the unborn baby, so when she eats, she must bear in mind that she is not eating for herself but for the baby as well and eating soil and drinking fizzy drinks with acid is not good for the baby. They would rather drink 100% juice; I cannot mention everything, or else others won't have anything to share.” FGD2, P2 (Facility 1)

“We explain to pregnant women that they must eat healthy food. We teach these women about the food they need to eat while pregnant….” P11, Mother mentor (Facility 7)

##### 3.1.2.6 Subtheme 3.2: Augmenting health literacy through supplementary information

When describing their role in supporting maternal nutrition among pregnant women, participants indicated that their responsibilities extend beyond providing nutritional advice and hygiene education. They reported engaging in activities aimed at enhancing health literacy through the provision of additional health-related information. This included information on the importance of early antenatal care booking, lifestyle practices during pregnancy, and safe sexual practices. CHWs and MMs described broadly similar roles in this area:

“We tell them not to drink or smoke, as that is not good for the baby.” FGD3, P5 (Facility 1)

“We advise them of the importance of following up with their check-ups at the clinic because the follow-ups are all important to check the development progress of the baby, and if they don't attend those check-ups, there might be undetected conditions which might lead to C-section delivery. In the past, we had a high death rate because the mothers were not following up on their consultation dates, and without consulting, they were not aware of the changes in their pregnancies. For example, the baby would be big and at birth, it would be difficult. However, if the pregnancy had been monitored, they would have known that the pregnancy needed a C-section and prepared in advance.” FDG5, P4 (Facility 6)

“We also advise them to attend antenatal classes early in pregnancy for the protection of the baby so that the baby is not infected.” FGD1, P3

“The mother should use a condom during intercourse because you might be intimate with a person, whilst you don't know if they are sick or not. Condoms help protect both the mother and the baby. If the mother is on medication, she should take her meds constantly so that she doesn't infect the baby.” FGD1, P2

“…We would show them the types of food they need to eat and also tell them to exercise that when they are pregnant, they are not disabled, they must be active instead of sleeping the whole day….” P4, Mother mentor (Facility 1)

The participants further indicated that their roles were not limited to maternal nutrition alone, but also to its comprehensiveness. They provided health education support to HIV-diagnosed patients.

“We give pregnant women health education when they come to the clinic, and we also offer them counselling to prepare them for HIV testing. After that, we give them counselling after testing whether they are positive or negative. We are not much into nutrition counselling, but we focus mostly on HIV counselling. We also connect them to momConnect.” P2, Mother mentor (Facility 2)

##### 3.1.2.7 Subtheme 3.3: Liaison with community and health services

Another role reported by the CHWs and MMs in supporting pregnant women with nutrition was liaising with communities and health services. The participants indicated that pregnant women facing challenges in accessing nutritious food were often referred to dieticians, nurses, and social workers. Participants also described linking some pregnant women to community-based support initiatives, such as food parcel programmes, to assist with household food needs:

“My sister let me say that we from Facility 1 are very lucky because when we visit house to house and find that the situation is bad. We come back to the clinic, and since the clinic is part of the church, they sometimes hand out food parcels to the less privileged. I informed my line manager that I am from a household that is not well, and she initiated handing out food parcels, as they don't have any other plan, and monthly she can assist, and we are grateful for the intervention.” FGD1, P6 (Facility1)

“We take the challenging matters to the nurses, and the nurse will attend to the matter and refer it to relevant professionals like social workers or dieticians.” P5, Mother mentor (Facility 3)

“…as a team we refer the people to the social workers and they social workers have their own programmes of handing out food parcels.” FGD1, P1 (Facility 1)

“We refer women who have problems with food to the sister, and the sisters will refer them to the dietitian in the clinic.” P3, Mother mentor (Facility 2)

## 4 Discussion

This study explored the understanding and perspectives of CHWs and MMs regarding maternal nutrition among pregnant women in Limpopo Province, South Africa. The findings indicate that while participants demonstrated awareness of the importance of maternal nutrition for maternal and foetal health, their knowledge varied, and their roles were shaped by broader contextual factors. These included cultural beliefs, socio-economic constraints, resource limitations, and misconceptions surrounding nutritional supplementation. Together, these findings highlight the complexity of achieving optimal maternal nutrition and the need for family-centred, contextually appropriate, and culturally responsive interventions.

Participants (both CHWS and MMs) identified maternal nutrition as important for foetal development, maternal immunity, and blood production, particularly in relation to iron- and vitamin-rich foods. These perspectives are consistent with existing evidence demonstrating that improved dietary quality during pregnancy is associated with positive birth outcomes, including reduced risk of adverse outcomes such as low birth weight and preterm birth (19). In support of this, similar findings have been reported in both high- and low-income settings, reinforcing the importance of adequate maternal nutrition across diverse contexts. A pooled analysis of European cohorts also showed that higher dietary quality was associated with a lower risk of delivering infants with low birth weight and small for gestational age (20). Another study in South Africa highlighted that increased intake of traditional dietary patterns with whole grains, legumes, vegetables, and traditional meats could reduce the odds of excessive weight gain (21).

In addition to diet, participants highlighted the role of physical activity as part of a healthy pregnancy. This aligns with evidence suggesting that healthy lifestyle behaviours during pregnancy, including physical activity, are associated with improved maternal and child health outcomes. The importance of a healthy lifestyle during pregnancy is well established in both higher- and lower-income countries. For instance, a study in high-income countries reported that healthy lifestyle behaviours during pregnancy have been linked with a lower risk of offspring becoming overweight or obese throughout childhood (22). This is reinforced by existing evidence across Africa that healthy lifestyle behaviours, such as physical activity, not only improve foetal outcomes but also maternal wellbeing (23). Although our findings align with evidence from both high- and low-income countries, existing literature indicates that, despite the promotion of healthy lifestyle behaviours through clinical guidelines and healthcare services, participation in physical activity remains low among pregnant women in South Africa (24).

Previous studies have reported that low participation in physical activity among pregnant women is associated with socio-economic constraints and limited time availability (25). Consistent with this evidence, the present findings highlight the importance of strengthening antenatal exercise promotion and physical health education within clinical settings. Such efforts should align with local guidelines, which emphasise safe physical activity during pregnancy, including engagement in light, home-based activities and the avoidance of heavy workloads to reduce the risk of pregnancy-related complications. Pregnant women's dietary practices are influenced by social and cultural norms. Participants reported that food choices during pregnancy are often shaped by family members, including grandparents, as well as friends and peers. These findings are consistent with those of Chakona and Shackleton, who showed that dietary practices among pregnant women are embedded within social contexts and cultural beliefs rather than being based solely on individual preferences. Such influences, including adherence to food taboos, have been associated with suboptimal dietary intake and may contribute to adverse pregnancy outcomes, including poor infant health (26).

Both CHWs and MMs further indicated that cultural practices influence not only dietary behaviours but also the utilisation of early antenatal care services among pregnant women. These findings suggest that household dynamics and social relationships play a role in shaping women's decision-making and perceived self-efficacy during pregnancy. This observation is supported by evidence from a study conducted in Bangladesh, which reported that factors such as knowledge, beliefs, social norms, and spousal support are important determinants of dietary practices among pregnant women. The study further showed that higher levels of knowledge, self-efficacy, and supportive household environments were associated with improved consumption of nutritious diets (27). A study conducted in Cape Town, South Africa by Chakona et al. (26) is consistent with findings from Bangladesh. These studies highlight the importance of understanding food taboos when designing culturally appropriate nutrition interventions aimed at addressing maternal and child malnutrition. In the present study, the findings further suggest that maternal nutritional practices are shaped by a range of interconnected factors, including cultural beliefs, social norms, and food taboos. These insights reinforce evidence from previous research indicating that interventions targeting maternal nutrition may benefit from adopting a family-centred approach (28). Evidence further indicates that interventions aimed at enhancing maternal self-efficacy, particularly in food-insecure households, are associated with improvements in maternal feeding behaviours (29). Interpreted within the framework of the Health Belief Model, the present findings suggest that CHWs and MMs recognise the benefits and importance of adequate nutrition during pregnancy (13). However, participants also described the presence of multiple perceived barriers, including cultural norms, poverty, and resource constraints, which may influence the adoption of recommended nutritional practices. Participants’ accounts of pregnant women hiding their pregnancy and delaying antenatal care reflect perceived susceptibility and perceived threat, with fears of bewitchment shaping both dietary and healthcare-seeking behaviour. Their reliance on personal experience and informal peer support when formal guidance was absent reflects the self-efficacy with which CHWs and MMs approach their role. The differing accounts participants gave of the advice they provided, for example, on physical activity, point to variation in the content and consistency of the information communicated to pregnant women.

From the perspectives of CHWs and MMs, perceived barriers and challenges appeared to affect the extent to which pregnant women could act on nutrition-related information, thereby shaping maternal dietary behaviours (13). Maternal health literacy and perceived dietary self-efficacy have been identified as important factors influencing pregnancy outcomes, including neonatal birth weight (30). Consistent with this, the present findings suggest that although CHWs and MMs demonstrated awareness of maternal nutrition and its importance, socio-economic conditions may have constrained the ability of pregnant women to translate this knowledge into practice.

These findings highlight the importance of considering the socio-economic context of pregnant women in the design of maternal nutrition interventions, particularly in disadvantaged settings. Addressing these contextual factors may support efforts to improve maternal dietary practices. Interpreted through the Health Belief Model, the findings indicate that behavioural change related to maternal nutrition is influenced by perceived barriers and challenges, including poverty, cultural norms, and limited access to resources. Targeted strategies that address these barriers may support improvements in dietary practices among pregnant women (13).

CHWs and MMs described their role as supporting pregnant women and communities through health education aimed at enhancing health literacy. This included guidance on recommended dietary practices, nutritional hygiene, self-care during pregnancy, and safe sexual practices. However, the findings also indicate that many CHWs and MMs had not received formal training in maternal nutrition. This may limit the scope and consistency of the information provided and reduce opportunities for effective preventive care.

Variations in knowledge related to maternal nutrition were observed across participants, suggesting differences in understanding and communication of key messages. These findings highlight the importance of strengthening capacity through standardised and ongoing training programmes. Such training should include maternal nutrition content, as well as communication, counselling, and community engagement skills, supported by access to evidence-based educational materials.

In addition, participants reported that some pregnant women relied on information from family members, peers, and social media. This pattern was described alongside concerns about limited trust in CHWs and MMs within communities. Participants indicated that some community members were hesitant to engage with their services. Existing evidence suggests that factors such as concerns about confidentiality and perceptions of professionalism may influence trust in CHW-delivered services (31). Addressing these factors through training, supervision, and community engagement strategies may support improved acceptance and utilisation of CHW- and MM-led maternal health services (31). Participants reported that some pregnant women rely on alternative sources of information, including family members, peers, and social media, for guidance on maternal nutrition. This pattern was described alongside concerns about limited trust in CHWs and MMs, with participants indicating that this may reduce engagement with the services and advice they provide. These findings are consistent with existing evidence suggesting that trust is a key factor influencing the uptake and effectiveness of community-based maternal and child health services (31).

Furthermore, participants highlighted the need to strengthen competencies related to ethical standards and professional practice. Previous research indicates that concerns related to confidentiality and perceived professionalism may influence community trust in CHWs, thereby affecting service delivery (31). Strengthening training, support, and supervision systems may enhance CHWs’ and MMs’ ability to navigate complex relationships within communities and the broader health system. In particular, context-specific training that includes confidentiality, respectful communication, and community engagement may support improved service acceptability and effectiveness. The findings also suggest that CHWs and MMs recognised the relationship between maternal nutrition and foetal growth and development. However, emphasis within participant accounts appeared to be more frequently placed on foetal outcomes than on maternal health. This pattern may reflect gaps in training and the framing of maternal nutrition messages. In addition, cultural practices were described as influencing both dietary behaviours and healthcare-seeking patterns among pregnant women. Participants indicated that these factors may shape food choices and access to services, including antenatal care. Taken together, these findings underscore the role of social, cultural, and relational factors, including trust, in shaping maternal nutrition practices and engagement with health services.

### 4.1 Strengths and limitations

Several limitations of this study should be acknowledged. First, focus group discussions with CHWs were conducted only in rural settings; therefore, the perspectives of CHWs working in urban areas may not be represented. This limits the ability to compare experiences across different settings. All CHWs and most MMs worked in rural settings, and no CHWs were recruited from urban areas. The findings, therefore, represent predominantly rural perspectives and should not be interpreted as a comparison between rural and semi-urban contexts. Second, although CHWs and MMs were sampled from facilities within the Capricorn District, the findings may not be generalisable to other regions, given the context-specific nature of qualitative research. Furthermore, the use of qualitative methods may have introduced social desirability bias, where participants could have underreported or selectively reported their experiences. Finally, the study sample comprised only female participants, which may have influenced the findings, as experiences and perceptions of maternal health can be shaped by gendered roles and lived experiences.

Despite these limitations, the study has several strengths. The inclusion of two participant groups, CHWs and MMs, provided complementary perspectives and enabled a more comprehensive understanding of maternal nutrition within community and healthcare contexts. This approach enhanced the depth and richness of the data and allowed for the exploration of diverse experiences and viewpoints.

In addition, the use of focus group discussions facilitated interactive dialogue among CHWs, encouraging the sharing of experiences and collective reflection. The study contributes context-specific insights from Limpopo Province, offering valuable evidence on the challenges and facilitators of achieving optimal maternal nutrition in a rural South African setting.

## 5 Conclusions

The findings of this study highlight the influence of cultural practices and beliefs, socio-economic constraints, limited resources, lack of trust, and misconceptions related to pregnancy supplements on maternal nutrition. These factors interact to shape maternal dietary behaviours and the extent to which nutrition-related information is adopted during pregnancy. The study further indicates that cultural norms and socio-economic challenges may limit the effectiveness of individual-level approaches, such as those focused solely on improving self-efficacy. Addressing maternal nutrition in this context requires approaches that account for the broader social and structural environment within which pregnant women make dietary decisions. Understanding these contextual factors within the setting of Limpopo Province provides important insights for the design and implementation of maternal nutrition interventions. Interventions should be context-specific and culturally responsive, considering the lived experiences of pregnant women as well as the roles of CHWs and MMs in supporting maternal health.

### 5.1 Recommendations

The findings underscore the need to strengthen the capacity of CHWs and MMs through standardised and comprehensive training. Such training should aim to ensure consistent, accurate, and evidence-based communication of maternal nutrition information to pregnant women. In addition, concerns related to community engagement and acceptability should be addressed. Strategies that enhance community awareness of the roles of CHWs and MMs, alongside efforts to strengthen trust and engagement, may improve the uptake of maternal health services and information.

## Data Availability

The raw data supporting the conclusions of this article will be made available by the authors without undue reservation.
